# Discovery of zirconium dioxides for the design of better oxygen-ion conductors using efficient algorithms beyond data mining†

**DOI:** 10.1039/c8ra02958j

**Published:** 2018-07-16

**Authors:** Joohwi Lee, Nobuko Ohba, Ryoji Asahi

**Affiliations:** Toyota Central R&D Laboratories, Inc. Nagakute Aichi 480-1192 Japan j-lee@mosk.tytlabs.co.jp

## Abstract

It is important to find crystal structures with low formation (*E*_v_) and migration-barrier (*E*_m_) energies for oxygen vacancies for the development of fast oxygen-ion conductors. To identify crystal structures with lower *E*_v_ and *E*_m_ than those of ground-state ZrO_2_, we first reoptimize the crystal structures of various oxides reported in the database, and then directly construct them using an evolutionary algorithm. For efficient searching, we employ the linearized ridge regression model for *E*_v_ using descriptors obtained from density functional theory calculations of the unit cells and apply the predicted *E*_v_ as a fitness value in the evolutionary algorithm. We also find a correlation between the *E*_v_ and *E*_m_ for the crystal structures of ZrO_2_. On the basis of this correlation, we confirm that the newly constructed crystal structures, as well as certain reoptimized structures from the database, that possess low *E*_v_ also have *E*_m_ lower than that of ground-state ZrO_2_. Our successful strategy consisting of a combination of the evolutionary algorithm, first-principles calculations, and machine-learning techniques may be applicable to other oxide systems in finding crystal structures with low *E*_v_ and *E*_m_.

## Abstract 2

Search for crystal structures of ZrO_2_ with higher oxygen-ion conductivity by evolutionary algorithms, first-principles calculations, and regression analysis.

## Graphical abstract

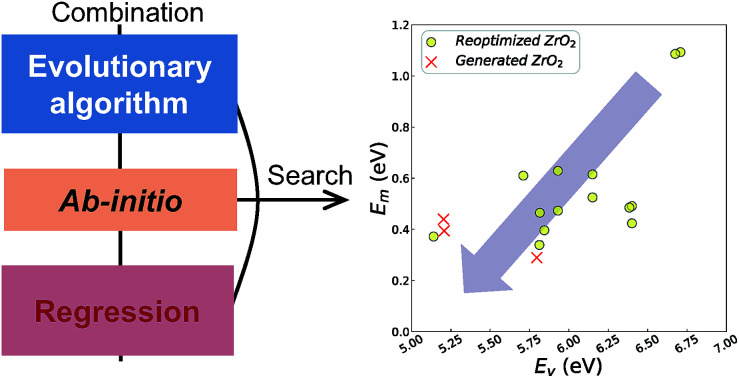

## 1. Introduction

A high oxygen ion conductivity (*σ*_O_) is an important property for applications^1,2^ such as the electrolytes of solid-oxide fuel cells (SOFC), oxygen separation membranes, and gas sensors. Low vacancy formation (*E*_v_) and migration-barrier (*E*_m_) energies of oxygen vacancies *V*_O_ are favorable for achieving a higher *σ*_O_.^3^ Currently, Y-doped ZrO_2_ (YSZ) is widely used because of its advantages such as abundance, chemical stability, non-toxicity, and low cost. This material shows the *σ*_O_ of ∼10^−2^ S cm^−1^ at the high temperature of 1000 K.^4^ For useful industrial applications, it is necessary to have similar *σ*_O_ values at lower temperatures or a higher *σ*_O_ at a similar temperature. Indeed, several oxides such as Gd-doped CeO_2_ (GDC),^5^ pure or Er-doped δ-phase Bi_2_O_3_,^6,7^ and Sr- and Mg-doped LaGaO_3_ (LSGM)^8,9^ have been reported to show higher *σ*_O_ than YSZ at the same temperature. However, the need for the development of new oxygen-ion conductors remains.

The type of crystal structure strongly affects the *σ*_O_ as well as *E*_v_ and *E*_m_. The monoclinic ground-state structure of ZrO_2_ (space group *P*2_1_/*c*) is known to have a low *σ*_O_. However, the *σ*_O_ can be significantly increased through phase transformation into either the tetragonal (space group *P*4_2_/*nmc*) or cubic fluorite structure (space group *Fm*3̄*m*) upon doping with Y.^5^ The δ-phase Bi_2_O_3_ in the defective fluorite structure (its high-temperature phase) is known to have a high *σ*_O_, whereas its α-phase counterpart is a low-temperature phase with a low *σ*_O_.^6^ Reported crystal structures with high *σ*_O_ are mainly fluorites, perovskites,^5^ and their derivatives, such as the defective fluorite and melilite^10^ structures. However, we expect that other undiscovered crystal structures may have high *σ*_O_ values.

The evolutionary algorithm is useful for the construction and search of crystal structures.^11^ This has been applied in combination with first-principles calculations for determining crystal structures with maximized or minimized functional properties (fitness values) such as enthalpy at high pressure,^12–14^ hardness,^15^ and dielectric constant.^16^ This algorithm is especially powerful for easily obtained fitness values, because it requires many individual crystal structures. From this perspective, the direct usage of the *E*_v_ or *E*_m_ as a fitness value may be inefficient because these properties should be obtained from supercell calculations.^17^ In this case, one useful method is machine-learning techniques.

A regression analysis, one of the machine-learning techniques, is used to predict a target variable on the basis of already accumulated data, which can be used for descriptors (also called “predictor variables” or “representations”). If the descriptors are composed of “density functional theory (DFT)-unit-cell descriptors” that contain information regarding the unit-cells obtained only from DFT calculations,^18,19^ the prediction model can be constructed with the advantage of computational efficiency. Some regression models have employed descriptors obtained from DFT calculations of the unit-cells to predict target variables such as the melting temperatures of binary metals,^20^ GW-level band gaps for inorganic compounds,^21^ and interatomic potentials.^22^ In addition, Deml *et al.*^23^ constructed a prediction equation for *E*_v_ using ordinary least-square regression (OLSR) with four unit-cell descriptors and 45 oxides. However, it is not yet guaranteed whether this prediction model maintains accuracy in predicting *E*_v_ for various types of crystal structures.

The purpose of the present study is to propose an efficient method to find various crystal structures with lower *E*_v_ and *E*_m_ values than those of ground-state ZrO_2_. To this end, we reoptimized the reported crystal structures of various oxides in the Materials Project Database (MPD),^24^ one of the inorganic materials databases based on first-principles calculations, and reconsidered their *E*_v_ and *E*_m_. We constructed a prediction model for *E*_v_ based on linearized regression analyses using DFT-unit-cell descriptors and subsequently constructed crystal structures based on the predicted *E*_v_ using the evolutionary algorithm. We also investigated the relationship between *E*_v_ and *E*_m_ for the crystal structures of ZrO_2_. Finally, we found crystal structures having *E*_v_ and *E*_m_ values lower than those of ground-state ZrO_2_.

## 2. Methodology

### 2.1. Overview

First, we reoptimized the crystal structures of several oxides from the MPD^24^ for ZrO_2_. The computed *E*_v_ and other properties of the unit-cells are used as the target variable and descriptors, respectively, for the regression analyses.

**Fig. 1:**
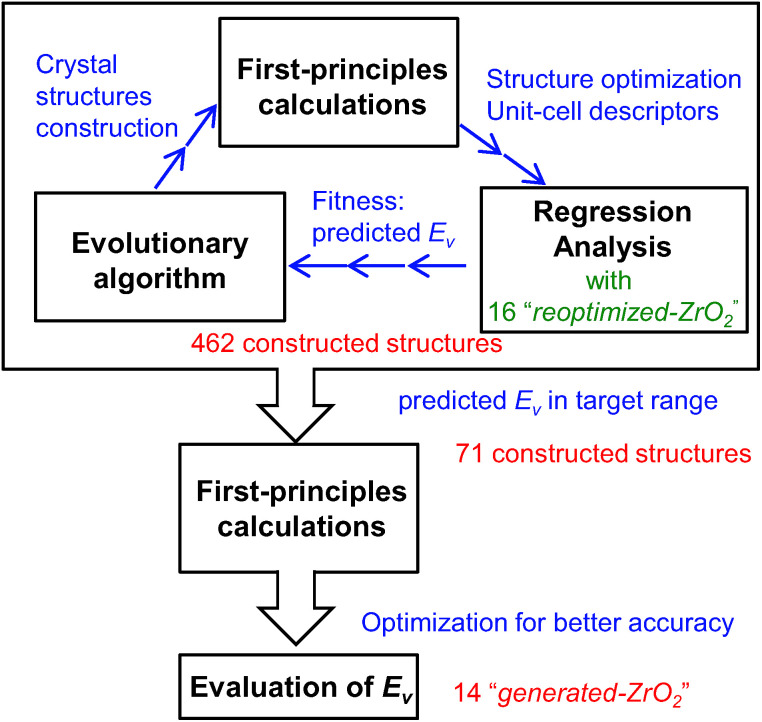
Flowchart of screening for crystal structures of ZrO_2_ with low *E*_v_ constructed by evolutionary algorithm and the regression analysis. The procedures in the large rectangular box are repeated to construct 462 crystal structures of ZrO_2_.

Fig. 1 shows the workflow for the construction of the crystal structures and prediction of the *E*_v_. New structures were constructed using the evolutionary algorithm. First-principles calculations were performed to optimize the constructed crystal structures and extract the DFT-unit-cell descriptors. The regression analyses were then applied to obtain the predicted *E*_v_. In total, 462 crystal structures were constructed through the repetition of cycles comprising the construction of crystal structures, calculation of DFT-unit-cell descriptors from the constructed structures, and application of regression analysis to predict *E*_v_.

We identified crystal structures with predicted *E*_v_ in the target range and performed structural optimization again under tighter conditions. The accurate *E*_v_ of the remaining crystal structures were computed from their supercells. Finally, we obtained 14 newly constructed crystal structures that have both the predicted and computed *E*_v_.

More details regarding each method are found in the next subsection.

### 2.2. Crystal structures for the training set in regression analysis

As mentioned in the previous subsection, we collected various crystal structures of oxides with cation-to-O ratios of 1 : 2 in the MPD^24^ for use as training data in regression analysis and for considering their *E*_v_ and *E*_m_. We set the number of atoms in the unit-cells to ≤30. We performed first-principles calculations for structural optimization and computation of the *E*_v_. To identify crystal structures with reliable *E*_v_, we adopted several filters. Firstly, the crystal structures must satisfy the convergence of electronic and ionic relaxations. Secondly, they should be non-metallic. Thirdly, they should maintain a displacement of the center of the *V*_O_ site of 0.1 Å at most after structural relaxations; this is for avoiding the convergence of crystal structures into other crystal structures. On the basis of these filters, we collected 16 crystal structures of ZrO_2_ with the computed *E*_v_, as summarized in Table 1. Henceforth, this set of 16 crystal structures is called “reoptimized-ZrO_2_” and each structure in the set is called by its space group type. Excluded crystal structures are summarized in Table S1 in ESI.† All the crystal structures in this study are displayed using the VESTA program.^25^

**Table 1:** Computed properties, including *E*_v_, for the 16 crystal structures of reoptimized-ZrO_2_

Space group type	Oxide reported in MPD^24^	*E* − *E*(ground-state) (eV per atom)	*E*_g_^GGA+U^ (eV)	Computed *E*_v_ (eV)^a^
*P*2_1_/*c*	ZrO_2_	0.000	3.53	6.15 (6.13, 6.16)
*Pbca*	ZrO_2_	0.007	3.52	6.15 (6.14, 6.15)
*I*4_1_/*amd*	ZrO_2_	0.013	3.89	6.40
*P*2_1_/*m*	TiO_2_	0.017	3.85	6.40 (6.38, 6.41)
*C*2/*c*	TiO_2_	0.018	3.85	6.38
*Pca*2_1_	ZrO_2_	0.019	3.78	5.93 (5.93, 5.93)
*P*4_1_2_1_2	SiO_2_	0.021	3.51	6.71
*P*4_2_/*mnm*	ZrO_2_	0.026	3.55	6.68
*Pnma*	TiO_2_	0.028	4.11	6.06 (6.01, 6.10)
*P*4_2_/*nmc*	ZrO_2_	0.029	3.89	5.81
*Pbcn*	ZrO_2_	0.032	3.85	5.82
*Fm*3̄*m*	ZrO_2_	0.037	3.38	5.84
*P*4/*n*	ZrO_2_	0.054	3.34	5.71 (5.35, 5.80)
*Pna*2_1_	SiO_2_	0.116	2.80	5.14 (4.97, 5.30)
*R*3̄	SiO_2_	0.126	3.75	5.93 (5.87, 6.00)
*P*6_3_*mc*	TiO_2_	0.179	3.75	6.51 (6.19, 6.76)

a Values in the parentheses are the minimum and the maximum values in sequence, which depend on the kinds of O sites.

### 2.3. Regression analysis

The linearized ridge regression (RR)^26^ model uses a minimization function of the OLSR with a L2-norm penalty term that is given by1
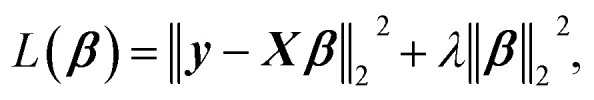
where ***β*** is an *n*-dimensional vector of the regression coefficients of the descriptors, ***X*** is an (*n* × *p*) descriptor matrix, ***y*** is an *n*-dimensional vector of the target property for the training set, and *λ* is a coefficient of the penalty term. Here, the penalty term is used for controlling the coefficients to avoid overfitting.

Table 2 shows the list of the DFT-unit-cell descriptors. To predict *E*_v_ of the crystal structures of ZrO_2_, RR was employed with all 11 descriptors. The OLSR with three descriptors (*x*_01_–*x*_03_), suggested by Deml *et al.*,^23^ was also used for comparison. Henceforth, the former and latter prediction models are referred to as RR-11-descriptors and OLSR-3-descriptors, respectively.

**Table 2:** DFT-unit-cell descriptors used for regression analyses

Name	Unit-cell-descriptor	Note
*x*_01_	Formation energy (Δ*E*_f_)	Relative energy from the Zr metal and the half-energy of O_2_ gas molecule
*x*_02_	Band-gap (*E*_g_^GGA+U^)	From the electronic DOS
*x*_03_	Center of O 2p-band (*E*_O2p_)	From the minimum energy of the electronic DOS to the VBM
*x*_04_	Volume per atom	
*x*_05_	Average bond length (*r*_Zr–O_)	Cutoff radius of 2.4 Å
*x*_06_	Coordination number of O (CN_O_)	Cutoff radius of 2.4 Å
*x*_07_	Mean of |*x*_02_ − *x*_02_(ground-state)|	
*x*_08_	Mean of |*x*_03_ − *x*_03_(ground-state)|	
*x*_09_	Mean of |*x*_04_ − *x*_04_(ground-state)|	
*x*_10_	Mean of |*x*_05_ − *x*_05_(ground-state)|	
*x*_11_	Mean of |*x*_06_ − *x*_06_(ground-state)|	

The training and test data were randomly divided in the ratio of 75% to 25%. To avoid overfitting, three-fold cross-validation (CV) was also used. The prediction errors were defined as root-mean-square-errors (RMSE) that were averaged for 30 different random samplings of the training set.

### 2.4. Structure construction using an evolutionary algorithm

We used the USPEX^11,27,28^ code to generate crystal structures based on an evolutionary algorithm. Here, the predicted *E*_v_ was used as a fitness value for minimizing the quantity |predicted *E*_v_ − 5 eV|. This form was applied to focus on the search for crystal structures with *E*_v_ values moderately lower than that (6.15 eV) of the ground-state *P*2_1_/*c* structure.

Each newly constructed unit-cell of ZrO_2_ was set to have four Zr and eight O atoms. Sixty first-generation crystal structures were produced using randomly selected space group types. The fitness value was obtained after structural relaxation based on the first-principles calculations. From the second generation onwards, new crystal structures were produced by genetic operators, namely, heredity (30%), random symmetric algorithm (30%), and mutation (40%). Each generation consisted of 40 crystal structures. The evolutionary algorithm was terminated if the best-ranked crystal structure was not changed over eight generations or after the number of generations reached 30.

### 2.5. First-principles calculations

All first-principles calculations were performed using the projector augmented-wave (PAW)^29,30^ method implemented in the Vienna Ab-initio Simulation Package (VASP)^31,32^ within the framework of the Generalized Gradient Approximation (GGA) of Perdew–Burke–Ernzerhof (PBE) form,^33^ including on-site Coulomb interaction^34^ with an effective U − J of 3 eV (ref. 35) (GGA+U) for the d-orbitals of Zr. Here, we employed the GGA+U rather than the GGA to decrease the number of metallic crystal structures constructed by the evolutionary algorithm (See Fig. S1 in ESI†). The valence electrons occupied the 4s, 4p, 5s, and 4d orbitals for Zr, and the 2s and 2p orbitals for O.

During the construction of crystal structures by the evolutionary algorithm, structural relaxations of the unit-cells (with the associated changes in lattice constants and atomic coordinates) were modeled until the interatomic force on each atom was reduced to within 0.01 eV Å^−1^. To avoid bad convergence, we constrained the number of ionic iteration cycles to the maximum of 120. The cutoff energy was set to 400 eV. The Brillouin zone was sampled by *Γ*-centered meshes with the density of 2π × 0.12 Å^−1^ for structural optimization such as fast screening. The calculations for the total energy and electronic density of states (DOS) of the optimized unit-cells were performed with a finer sampling of 2π × 0.08 Å^−1^ of the Brillouin zone. Subsequently, structural optimizations were performed for the survivors of the evolutionary algorithm again, but in tighter computational conditions: the cutoff energy was increased to 500 eV, and the Brillouin zone was sampled using *Γ*-centered 8 × 8 × 8 meshes.

The computed *E*_v_ were obtained using the supercell method^17^ as follows,2
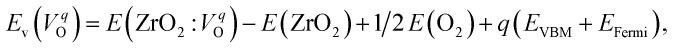
where 
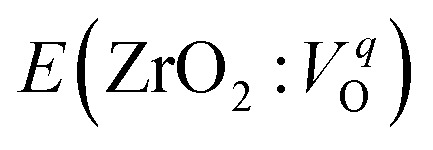
 is the energy of a supercell including a *V*_O_, *E*(ZrO_2_) is the energy of a supercell of pure ZrO_2_, 1/2*E*(O_2_) is the half-energy of an O_2_ gas molecule (used as the chemical potential of O for the O-rich condition), *q* is the unit charge, *E*_VBM_ is the energy of the valence band maximum (VBM), and *E*_Fermi_ is the Fermi level, that is a variable changes between the VBM and the conduction band minimum (CBM). The chemical potential of O depends on the processing condition, but it provides a constant shift to the *E*_v_ of all of the crystal structures of ZrO_2_ and the general consequence related to the ranks of the *E*_v_ are unchanged. Therefore, we only considered the O-rich condition in this study (See other O-rich conditions in Table S2 in ESI†). We calculated both of the *E*_v_ for a neutral oxygen vacancy 
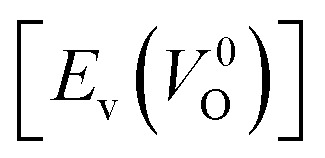
 and a doubly charged oxygen vacancy 
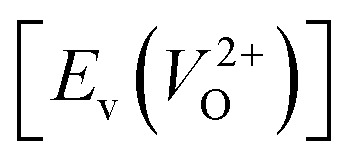
. For a prediction model based on the evolutionary algorithm and regression analysis, 
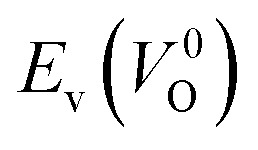
 was used as the computed *E*_v_. The calculations for the supercell with a *V*_O_ were performed until the interatomic forces on each atom were reduced to within 0.02 eV Å^−1^ with fixed lattice constants. The sizes of the supercells were set such that the lengths of the three lattice constants were ∼10 Å. *Γ*-centered 2 × 2 × 2 meshes were used to describe the *k*-space. The types of O sites were confirmed by SPGLIB implemented in PHONOPY.^36,37^ For a crystal structure containing more than two types of O sites, the computed *E*_v_ values were averaged.

**Fig. 2:**
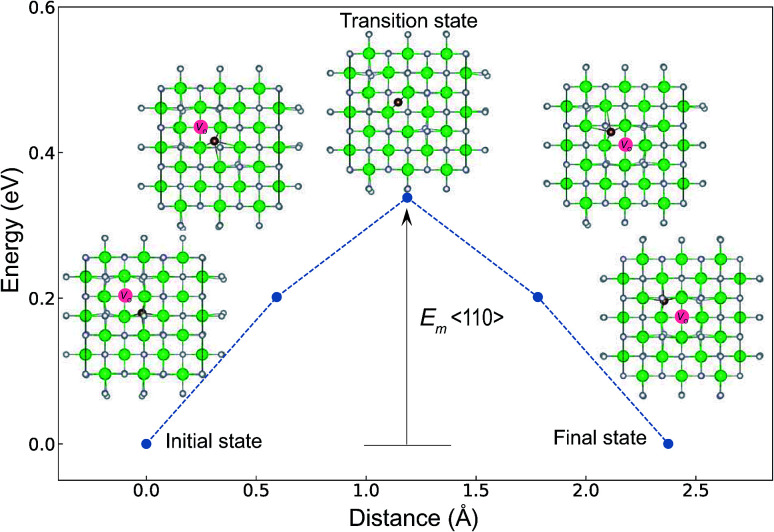
Energy differences of the intermediate states (images) of the *P*4_2_/*nmc* structure of ZrO_2_, which were obtained by the CI-NEB method. The atomic coordinates in the figure were shown from the normal direction to the (001) plane. Green and light gray spheres denote Zr and O atoms, respectively. A brown sphere is used to emphasize the migrating O atom along the 〈110〉 path. A pink circle denotes a *V*_O_ site. The *V*_O_ at the initial or final state is formed by removing an O atom and breaking four O–Zr bonds. The *E*_m_ is defined as the energy difference between the transition state and the initial or final state. The migrating O atom has four O–Zr bonds at the initial state, while it maintains only two O–Zr bonds at the transition state and breaks the other two O–Zr bonds.

The minimum energy path (MEP) for a *V*_O_ migration was obtained by the climbing-images nudged-elastic-band (CI-NEB) method.^38,39^ The *E*_m_ of a *V*_O_ can be defined as the energy difference between the transition state and the initial or final state from the obtained MEP, as shown in Fig. 2. The CI-NEB calculations were performed with three intermediate images (states) until the forces decreased below 0.03 eV Å^−1^ with a spring constant of 5 eV Å^−2^ between images. Here, the climbing images with even numbers were used to increase the probability of finding the transition state. We employed a 
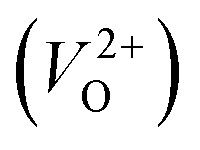
 for *E*_m_, assuming that *V*_O_ were formed by the reduction of cation valency upon doping. The supercells with a 
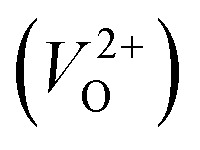
 were described by decreasing two electrons of the background charge for compensation. The internal coordinates for the supercells with a 
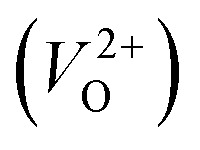
 were relaxed in the same computational conditions as those for 
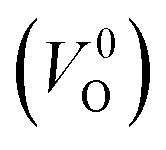
.

## 3. Results and discussion

### 3.1. Construction of a prediction model for *E*_v_ using DFT-unit-cell descriptors

As mentioned in the Introduction, Deml *et al.*^23^ constructed a linear prediction model for the *E*_v_ with four unit-cell descriptors, namely, the formation energy of oxides (Δ*E*_f_) obtained as the fitted elemental-phase reference energy (FERE),^35^ the computed band-gaps obtained by GGA+U (*E*_g_^GGA+U^) or GW^40^ calculations, the O 2p band center (*E*_O2p_), and the differences in electronegativity between the cations and O, for 45 different oxides in six crystal structures. Their prediction error was reported as ∼0.20 eV as the mean absolute error (MAE). To evaluate whether this model is well-fitted for the different crystal structures upon fixing the substituted chemical elements and composition ratio as listed in Table 1, we employed the OLSR-3-descriptors model with the descriptors *x*_1_–*x*_3_ listed in Table 2. For the RR-11 descriptor, the average difference in electronegativity between the cations and O is not used because it does not vary among the different types of crystal structures. The 16 crystal structures of the reoptimized-ZrO_2_ in Table 1 were used to construct the prediction model.

**Fig. 3:**
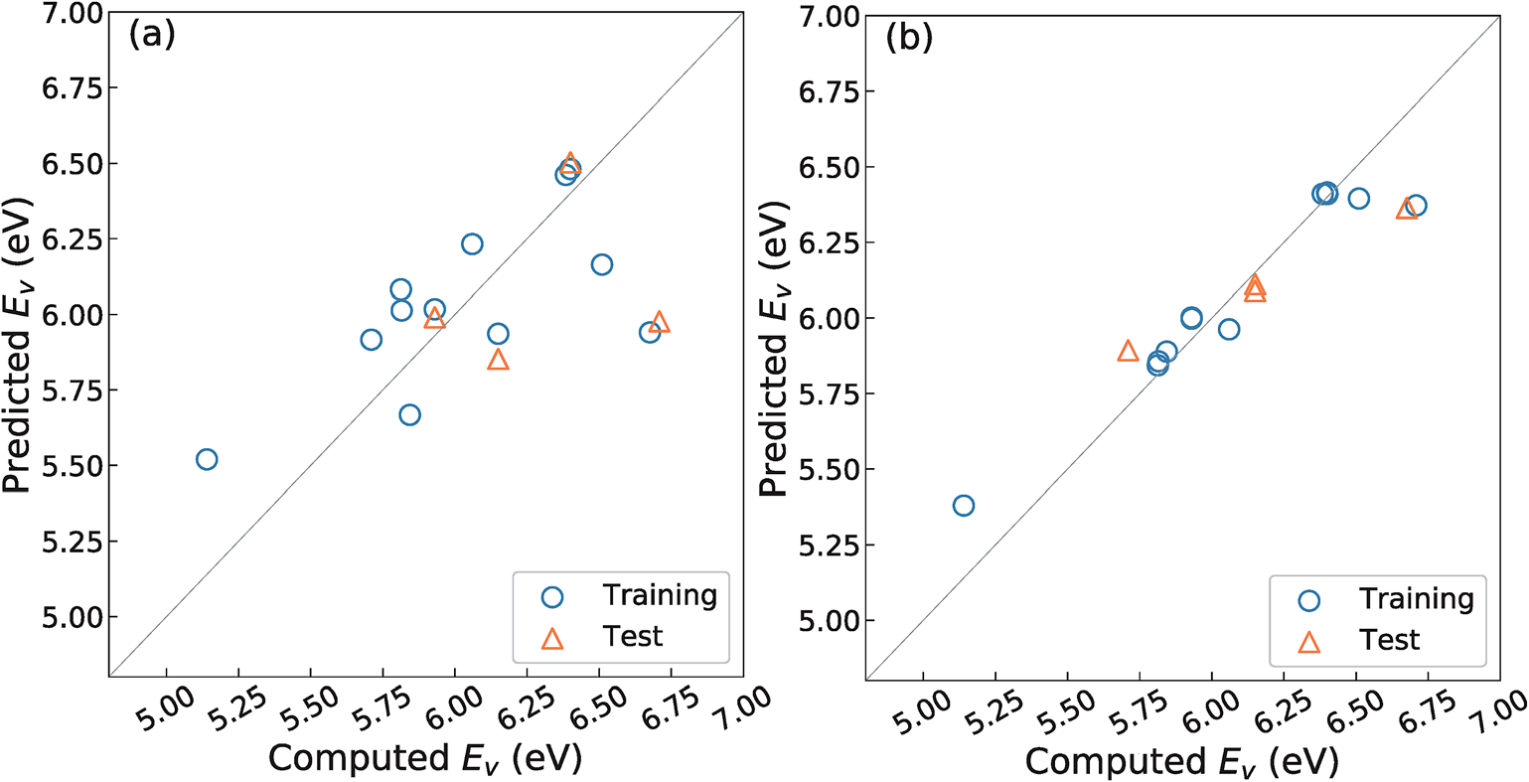
Relationship between the computed and predicted *E*_v_ by using regression analyses with the 16 crystal structures of reoptimized-ZrO_2_ with (a) 3-OLSR-descriptors and (b) 11-RR-descriptors. One snapshot is drawn from thirty repeated random samples for training and test data. The RMSE of test data averaged from thirty samplings from the prediction models using (a) the 3-OLSR-descriptors and (b) the 11-RR-descriptors are 0.51 and 0.16 eV, respectively.

Fig. 3(a) shows the relationship between the predicted and computed *E*_v_ obtained by using the OLSR-3-descriptors model. The distribution of data is largely scattered from the diagonal line that indicates equality between the predicted and computed *E*_v_. As arranged in Table 3, the CV-score and RMSE of the test set are 0.76 and 0.51 eV, respectively, which are much larger errors than the MAE of 0.20 eV for the 45 oxides.^23^ The large difference between the CV-score and RMSE of the test set also indicates that the prediction model has a large dependence on the type of training set. Therefore, it implies that the prediction model using the OLSR-3-descriptors is not good enough to predict the *E*_v_ of the various crystal structures of ZrO_2_.

**Table 3:** Errors between the predicted and computed *E*_v_ of two regression analyses using the 16 crystal structures of reoptimized-ZrO_2_. The prediction error values are obtained as RMSE in eV. The parentheses denote the standard deviation of values averaged over thirty readings

Prediction Model	Data divided into training (75%) and test (25%) set			Whole data	
	RMSE of training set	CV-score	RMSE of test set	RMSE of training set	CV-score
OLSR-3-descriptors	0.27 (0.05)	0.76 (0.36)	0.51 (0.22)	0.31	0.60 (0.19)
RR-11-descriptors	0.04 (0.04)	0.17 (0.06)	0.16 (0.16)	0.02 (0.01)	0.14 (0.07)

To improve the prediction accuracy, we constructed RR-11-descriptors by adding more DFT-unit-cell descriptors on top of the three descriptors; these DFT descriptors are intuitively thought to be related to the forming and breaking of chemical bonds. We employed the RR model, which can control the coefficients of various descriptors through a penalty term and thereby minimize overfitting. Fig. 3(b) shows the relationship between the predicted and computed *E*_v_ obtained by using the RR-11-descriptors model. The scattering of points from the diagonal line is significantly decreased. The CV-score and RMSE of the test sets are 0.17 and 0.16 eV, respectively, which are much smaller than those obtained from the OLSR-3-descriptors model. The similarity in the two values suggests the absence of any serious overfitting.

Furthermore, we applied the RR-11-descriptor model with a training set consisting of all 16 crystal structures of reoptimized-ZrO_2_. In the results, the CV-score slightly decreased to 0.14 eV. This improvement can be attributed to the increased number of training data points. This value is also only 0.02 eV different from that of the RMSE of the test set of the prediction model with a 75% training set, which suggests the absence of any serious overfitting. Finally, we identified the coefficients for a linear fitting function applied as the fitness value in the evolutionary algorithm by using the RR-11-descriptors model with all 16 crystal structures of the reoptimized-ZrO_2_.

### 3.2. Crystal structures with low *E*_v_

As Fig. 1 shows, we generated 462 crystal structures through combinations of the regression analysis for the *E*_v_ and evolutionary algorithm, and screened out 71 crystal structures based on the criterion |predicted *E*_v_ − 5| < 0.5 eV. After crystal relaxation once again under tighter conditions, we obtained 14 remaining crystal structures with the predicted and computed *E*_v_, excluding those that were metallic or could not satisfy the criteria for structural optimization. The detailed properties of the 14 crystal structures are listed in Table 4. Henceforth, this set of crystal structures is referred to as “generated-ZrO_2_”, and the name of each such structure is prefixed with “*Gen*-” (“generated or constructed”); these structures are numbered in the order of the computed *E*_v_. These crystal structures have very low symmetry; most of them are *P*1. To identify similar crystal structures with higher symmetry, a looser tolerance for finding the space group was also applied.

**Table 4:** Computed properties of the 14 crystal structures of generated-ZrO_2_

Name of constructed crystal structure	Space group type^a^	*E* − *E*(ground-state) (eV per atom)	*E*_g_^GGA+U^ (eV)	Predicted *E*_v_ (eV)	Computed *E*_v_ (eV)^b^
*Gen*-01	*P*1 (*Pnma*)	0.113	2.77	5.11	5.11 (4.94, 5.30)
*Gen*-02	*Pnma* (*Pnma*)	0.110	2.82	5.18	5.21 (5.11, 5.30)
*Gen*-03	*P*1 (*Pnma*)	0.110	2.83	5.18	5.21 (5.11, 5.30)
*Gen*-04	*P*1̄ (*Pnma*)	0.110	2.83	5.18	5.21 (5.11, 5.30)
*Gen*-05	*P*1 (*Pnma*)	0.110	2.83	5.18	5.21 (5.11, 5.31)
*Gen*-06	*P*2_1_2_1_2_1_ (*Pnma*)	0.110	2.83	5.18	5.21 (5.11, 5.30)
*Gen*-07	*Pca*2_1_ (*P*6_3_*mc*)	0.223	2.86	5.80	5.69 (5.56, 5.83)
*Gen*-08	*P*1 (*P*4_2_/*nmc*)	0.024	3.79	5.80	5.79 (5.79, 5.79)
*Gen*-09	*P*1 (*P*4_2_/*nmc*)	0.023	3.97	5.75	5.85 (5.85, 5.85)
*Gen*-10	*P*1 (*P*2_1_/*m*)	0.021	4.20	5.85	6.03 (5.90, 6.16)
*Gen*-11	*P*1 (*P*2_1_/*m*)	0.021	4.20	5.86	6.03 (5.90, 6.17)
*Gen*-12	*P*1̄ (*P*2_1_/*m*)	0.021	4.18	5.86	6.03 (5.90, 6.17)
*Gen*-13	*P*1 (*Fdd*2)	0.030	3.44	6.00	6.20 (6.07, 6.33)
*Gen*-14	*P*1 (*P*2_1_/*m*)	0.113	3.70	6.53	6.39 (6.33, 6.53)

a The space group in parentheses is identified using looser tolerance in SPGLIB in PHONOPY code^36,37^ to determine similar higher-symmetry crystal structures.

b Values in the parentheses are the minimum and the maximum values in sequence, which depend on the kinds of O sites.

**Fig. 4:**
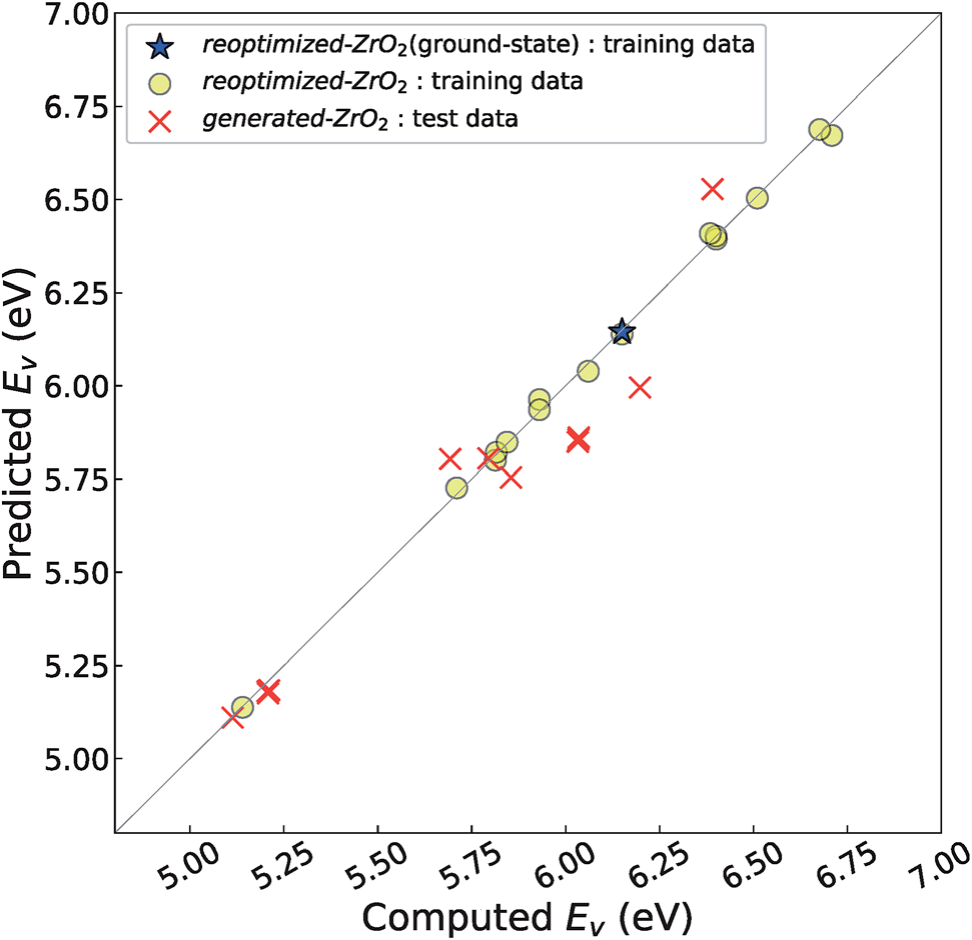
Relationship between the computed and predicted *E*_v_ of the training- and test-data crystal structures. The crystal structures for the training and test data are from reoptimized-ZrO_2_ and generated-ZrO_2_, respectively. The predicted *E*_v_ is predicted by the 11-RR-descriptors model. The RMSE of the 14 test-data crystal structures is 0.11 eV. Considering that the three-fold CV-score for the 16 training data is almost the same as 0.14 eV, overfitting is avoided.

Fig. 4 shows the relationship between the computed and predicted *E*_v_ of the crystal structures of reoptimized-ZrO_2_ and generated-ZrO_2_, which are used as the training and test data, respectively. The *E*_v_ of the ground-state *P*2_1_/*c* structure is emphasized with a star-shaped mark for easier viewing. Note that the distribution of the predicted *E*_v_ for the 14 test-data crystal structures is in the range 5.11–6.53 eV, which is wider than the screening criteria. This is because some crystal structures are relaxed into more stable structures, with changes in the predicted *E*_v_.

The distribution of the test data is close to the diagonal line. The prediction error is only 0.11 eV, which is almost the same as the CV-score of the RR-11-descriptors model with the 16 training data (0.14 eV). This means that the *E*_v_ of the 14 crystal structures of generated-ZrO_2_ have been successfully predicted using only DFT-unit-cell descriptors. Note that the distribution of the 16 training data points is very close to the diagonal line in Fig. 3(b), because their predicted *E*_v_ are obtained as the training data, and not as the test data.

**Fig. 5:**
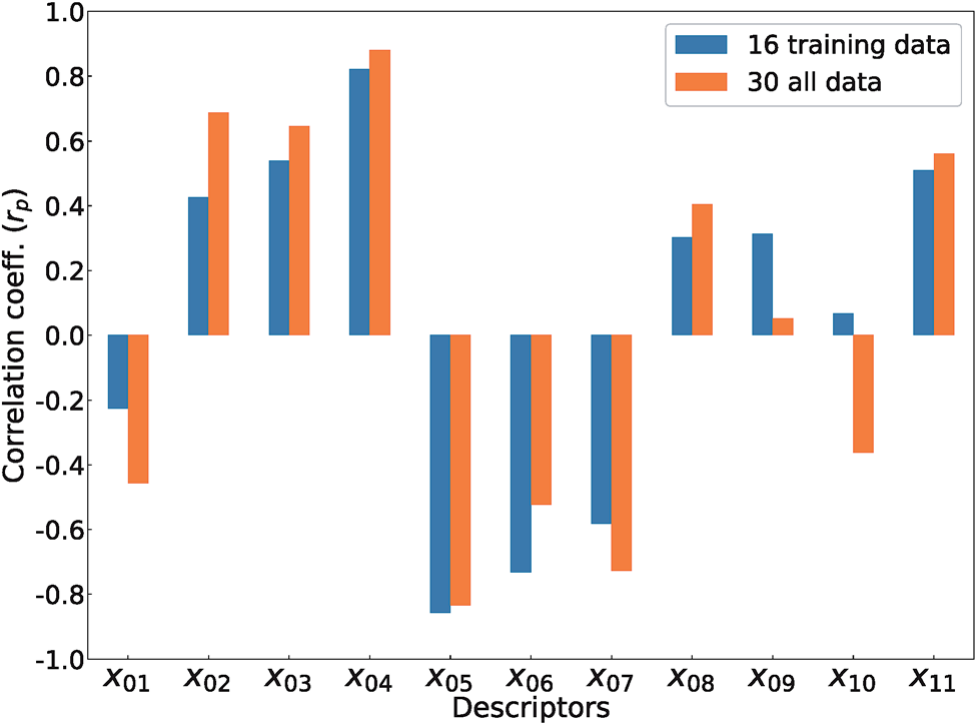
Pearson's linear correlation coefficients (*r*_p_) between the computed *E*_v_ and the 11 DFT-unit-cell descriptors for the 16 training data points (reoptimized-ZrO_2_) and the 30 total data points (the crystal structures of reoptimized-ZrO_2_ and generated ZrO_2_).

Fig. 5 shows Pearson's linear correlation coefficient (*r*_p_) for each DFT-unit-cell descriptor with respect to the computed *E*_v_. The absolute value of *r*_p_ is larger than 0.7, which indicates a strong correlation between the two properties for a given data point.^41^ For the 16 crystal structures of reoptimized-ZrO_2_, the |*r*_p_| for the volume per atom, average bond lengths (*r*_Zr–O_), and O coordination number (CN_O_) are 0.82, 0.85, and 0.73 (high), respectively, while the |*r*_p_| for the formation energy (Δ*E*_f_) and *E*_g_^GGA+U^ are 0.22 and 0.42 (low), respectively. This observation differs from that of Deml's model^23^ for 45 various oxides, which showed strong correlations between the FERE and *E*_v_, and between the *E*_g_^GGA+U^ and *E*_v_, with |*r*_p_| values of 0.84 and 0.89, respectively. The different accuracies of the prediction models between this study and ref. 23 can arise from several factors. Firstly, the 45 training-data oxides used in ref. 23 included six types of crystal structures with high symmetries, namely, anti-fluorite, fluorite, rocksalt, rutile, perovskite, and spinel. Most of these oxides are energetically stable for various combinations of constituent elements. However, the crystal structures of ZrO_2_ in this study all have different types of metastable lower-symmetry crystal structures. The contribution of each descriptor in the regression analysis may differ. Secondly, the range of training data can also affect the accuracy of prediction model. The *E*_v_ of the 45 training-data structures in ref. 23 are widely distributed between 2.6 and 7.0 eV, whereas the *E*_v_ of ZrO_2_ in this study are distributed between only 5.1 and 6.7 eV.

The *r*_p_ changes for all 30 crystal structures of the reoptimized-ZrO_2_ and generated-ZrO_2_. The values of |*r*_p_| for the volume per atom and *r*_Zr–O_ are similarly large as 0.88 and 0.82 respectively, while |*r*_p_| for CN_O_ decreases to 0.49. The |*r*_p_| for Δ*E*_f_ and *E*_g_^GGA+U^ changed to 0.51 and 0.69, respectively. When the RR-11-descriptors model is applied to all 30 crystal structures, the prediction accuracy is improved, with the RMSE of the test data of 0.08 eV compared with that of the same prediction model (0.16 eV) with the 16 training data crystal structures. Because the descriptors also maintain their correlations with each other in the regression analysis, the important unit-cell descriptors for the prediction of *E*_v_ can be changed according to the target materials and training data. Therefore, we suggest employing more types of unit-cell descriptors but with regression analysis using a penalty term; this is a useful way to construct the prediction model of the *E*_v_.

We identify many crystal structures having *E*_v_ lower than that of the ground-state *P*2_1_/*c* structure. As shown in Table 1 and Fig. 4, eight crystal structures, including the *P*4_2_/*nmc* and *Fm*3̄*m* structures that are well-known high-temperature structures of ZrO_2_ and that can be stabilized by extrinsic doping,^5^ reveal lower *E*_v_ than that of the ground-state *P*2_1_/*c* structure. In particular, the *Pna*2_1_ structure, which is obtained from the structural data of SiO_2_, shows the lowest *E*_v_ of 5.14 eV, which is almost 1 eV less than that of the ground-state *P*2_1_/*c* structure. This implies that exchanging the substituting elements in the crystal structures of other oxides can be an efficient way to reconsider a functional property. Additionally, we obtain 14 crystal structures based on the evolutionary algorithm that have lower *E*_v_ than that of the ground-state *P*2_1_/*c* structure. This suggests that the discovery of a crystal structure with a special functional property can be accelerated by a combination of the machine-learning technique and the direct construction of crystal structures based on the evolutionary algorithm.

Meanwhile, we reveal why the neutral oxygen vacancy 
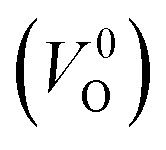
 is mainly employed in the calculations of the *E*_v_ for the prediction model. The *V*_O_ in the crystal structures of ZrO_2_ has been widely investigated. The *P*2_1_/*c* and *P*4_2_/*nmc* structures are known to form a deep level inside the *E*_g_ based on the GGA^42–44^ and HSE06 (ref. 45 and 46) calculations. Additionally, the *E*_v_ of the 
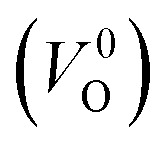
 in the dilute limit is physically well defined regardless of the doping level of the cation sites, and quickly converged with respect to the supercell size.

**Fig. 6:**
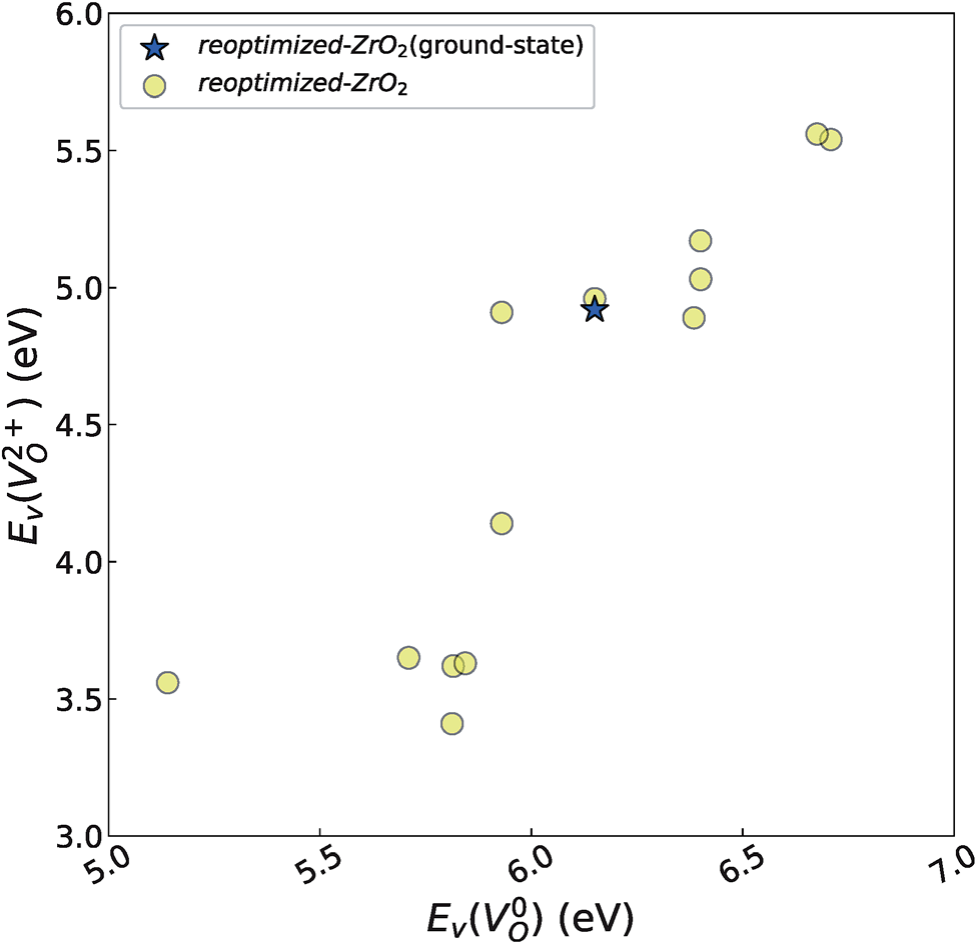
Relationship of the computed 
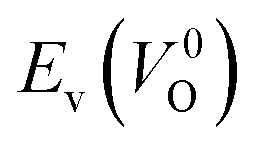
 and 
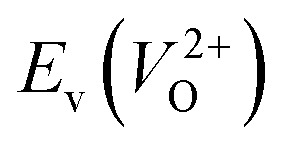
 of the 14 crystal structures of reoptimized-ZrO_2_. The *r*_p_ between the 
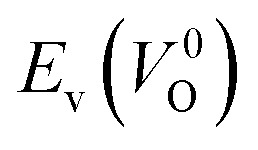
 and 
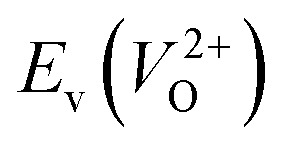
 is 0.86, which implies a very strong correlation between the two properties.

However, it is also worth investigating the relationship between 
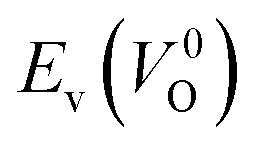
 and 
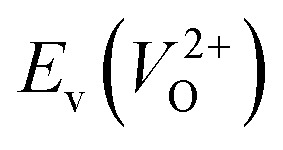
 considering the cases of extrinsic doping by aliovalent ions. Here, for 
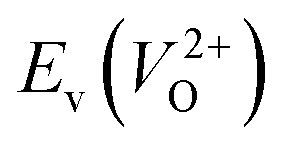
, we fixed the *E*_Fermi_ at the center of the *E*_g_ because the crystal structures of reoptimized-ZrO_2_ are all insulators (*E*_g_^GGA+U^ > ∼3 eV). Among the 16 crystal structures of the reoptimized-ZrO_2_ in Table 1, the *Pnma* and the *P*6_3_*mc* structures were excluded from the computation of 
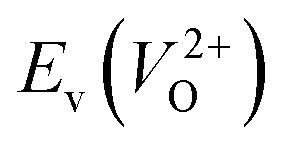
 because crystal structures with a 
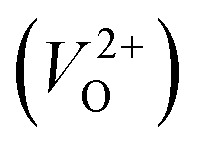
 are considered too unstable for convergence. Fig. 6 shows the relationship between the computed 
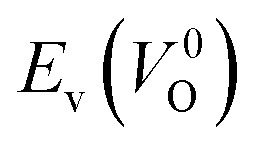
 and 
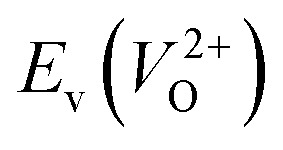
 of the 14 crystal structures of the reoptimized-ZrO_2_. It is noticeable that the 
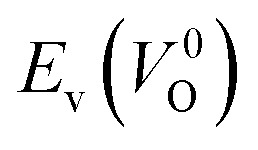
 and 
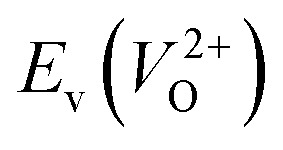
 have a very strong correlation with the *r*_p_ of 0.86. This suggests that the crystal structures with low 
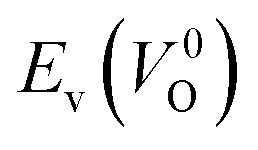
 also have low 
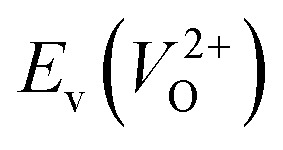
, which implies that extrinsic doping with aliovalent ions may also more easily generate *V*_O_ for these crystal structures (numerical values are summarized in Table S3 in ESI†).

Before proceeding, it is worthwhile to discuss the comparison of the computed values (*E*_g_ and *E*_v_ shown in Fig. S1 in ESI†) between the GGA+U and GGA methods. It is noticeable that the *E*_g_ obtained by the GGA+U are larger than those by the GGA, which implies that the GGA+U is useful to reduce the underestimation of the *E*_g_. For the computed *E*_v_, it is found that the *E*_v_ obtained by the GGA are slightly smaller or almost the same as those obtained by the GGA+U. This suggests that the GGA+U does not lose its reliability compared with the GGA, despite including the empirical U parameter.

### 3.3. Crystal structures with low *E*_m_

We summarize the *E*_m_ values of the *Fm*3̄*m* and the *P*4_2_/*nmc* structures obtained herein and in former computational studies^42,47–50^ in Table 5. Former computational studies have mainly used the GGA, rather than the GGA+U. To compare the results, we also employ the GGA with the PBE form.^33^ The *E*_m_ values obtained herein using the GGA are almost the same as those mentioned in previous computational reports. The *E*_m_ values obtained using the GGA+U are larger than those obtained using the GGA for both the *Fm*3̄*m* and *P*4_2_/*nmc* structures.

**Table 5:** *E*_m_ values of the *Fm*3̄*m* and the *P*4_2_/*nmc* structures

Crystal structure	*E*_m_ (eV)^a^		Reference	Method	GGA parameterization
	From GGA	From GGA+U			
*Fm*3̄*m*	0.26 (0.54)	0.39 (1.80)	This study	PAW (VASP code)	Perdew *et al.* (PBE)^33^
	0.25 (0.45)		47	Atomic orbital (SIESTA code)^63^	Perdew *et al.* (PBE)^33^
	0.24		48	PAW (VASP code)	Perdew *et al.* (PBE)^33^
	0.26 (0.54)		49	PAW (VASP code)	Perdew *et al.* (PBE)^33^
	0.28		50	Plane-wave with US-PP^d^ (Quantum-espresso code)^64^	Perdew *et al.* (PBE)^33^
*P*4_2_/*nmc*	0.26 (1.44)^b^, 0.63 (1.57)^c^	0.34 (1.74)^b^, 0.64 (1.66)^c^	This study	PAW (VASP code)	Perdew *et al.* (PBE)^33^
	0.22 (1.35)^b^, 0.61 (1.43)^c^		42	PAW (VASP code) with cutoff energy of 250 eV	Perdew *et al.*^65^

a Values outside and in the parentheses are obtained using
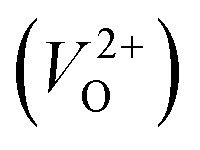
 and 
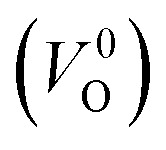
, respectively.

b (110) direction in a tetragonal cell.

c (001) direction in a tetragonal cell.

d Ultrasoft pseudopotentials.^66^

We also compare the *E*_m_ values of 
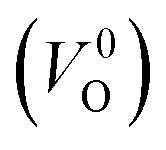
 and 
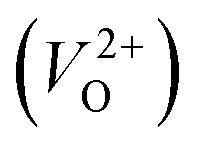
. Table 5 shows that the *E*_m_ values of 
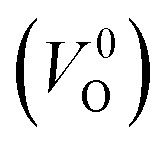
 are much larger than those of 
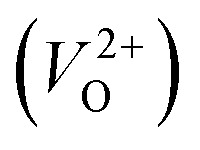
, independent of the crystal structure and the exchange-correlation functional. Larger *E*_m_ values for 
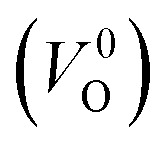
 than for 
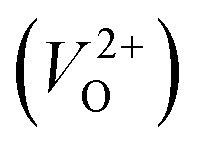
 have been reported in other oxides like MgO^51^ and ZnO.^52^ When a *V*_O_ jumps into its nearest-neighboring O site, the O atom at the nearest-neighboring site migrates in the opposite direction. For 
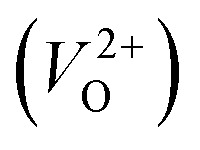
, no occupied levels are present inside *E*_g_. On the other hand, for 
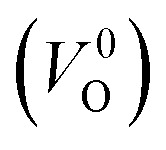
, the two electrons occupying the deep level have transition into the conduction band when the moving O atom is in the transition state (center of the hopping path),^47^ which may need more energy for *V*_O_ hopping. Considering the abovementioned reasons, we employed 
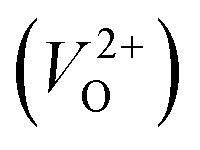
 for the *E*_m_ in this study.

Hereafter, we will refer to the *E*_m_ obtained by the GGA+U calculations.

In the case of the initial-state structure for the CI-NEB method, we employed the relaxed supercell possessing a 
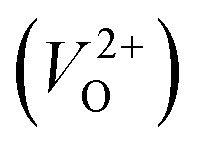
 having the lowest energy among the different O sites. For the 14 crystal structures of reoptimized-ZrO_2_ that have well-converged structures with a 
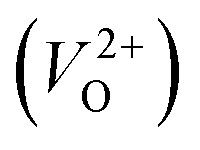
, we considered various final-state structures that have the *V*_O_ at the nearest-neighboring O site with the cutoff distance of 3.5 Å for the CI-NEB method (see Fig. S2 in ESI†). Among the *E*_m_ values of different migration paths, the lowest value was extracted; we then checked whether the *V*_O_ could migrate to the original site in the nearest-neighboring supercell *via* the one type of migration path with the lowest *E*_m_, because the migration of the *V*_O_ through the bulk system is practically meaningful. In 12 of the 14 crystal structures, *V*_O_ could migrate to the original site in the nearest-neighboring supercell *via* the migration path with the lowest *E*_m_. However, for the *P*4/*n* and *R*3̄ structures, a *V*_O_ needs different paths with different *E*_m_ values to migrate to the original site in the nearest-neighboring supercell. We define the *E*_m_ for these structures as the minimum barrier energy required for migration from the original site to that in the nearest-neighboring supercell with several kinds of CI-NEB calculations. The energy diagrams for the migration of a *V*_O_ are shown in Fig. S3 and Table S4 in ESI.†

**Fig. 7:**
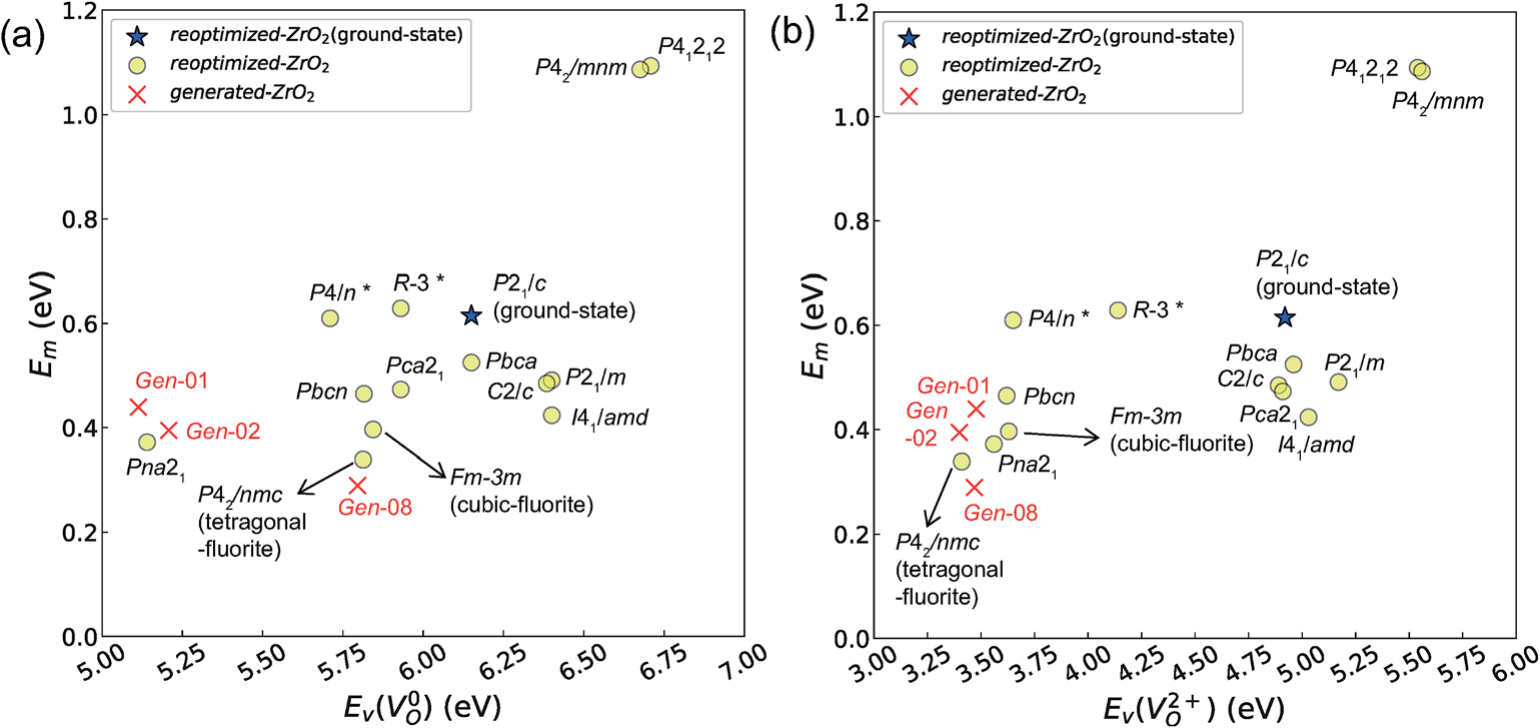
Relationship between the computed *E*_v_ [(a) 
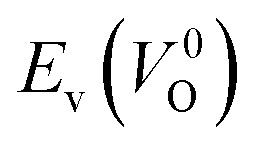
 and (b) 
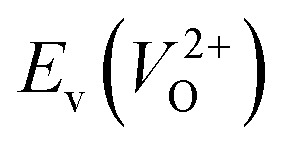
, respectively] and *E*_m_ of various crystal structures of ZrO_2_. The *r*_p_ between the *E*_m_ and 
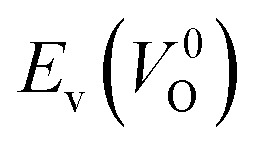
 and between the *E*_m_ and 
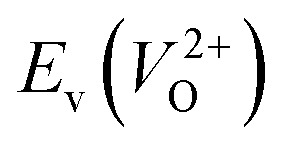
 of the 14 crystal structures from reoptimized-ZrO_2_ are 0.64 and 0.62, respectively, which implies that the correlation in a reasonable level is found. The crystal structures with asterisks (*) need several different types of migration for a *V*_O_ to move to the same site in the nearest-neighboring computational cell (see Fig. S3 in ESI†). On the basis of this information, the *E*_m_ of three crystal structures from generated-ZrO_2_ are obtained (red × marks).

**Fig. 8:**
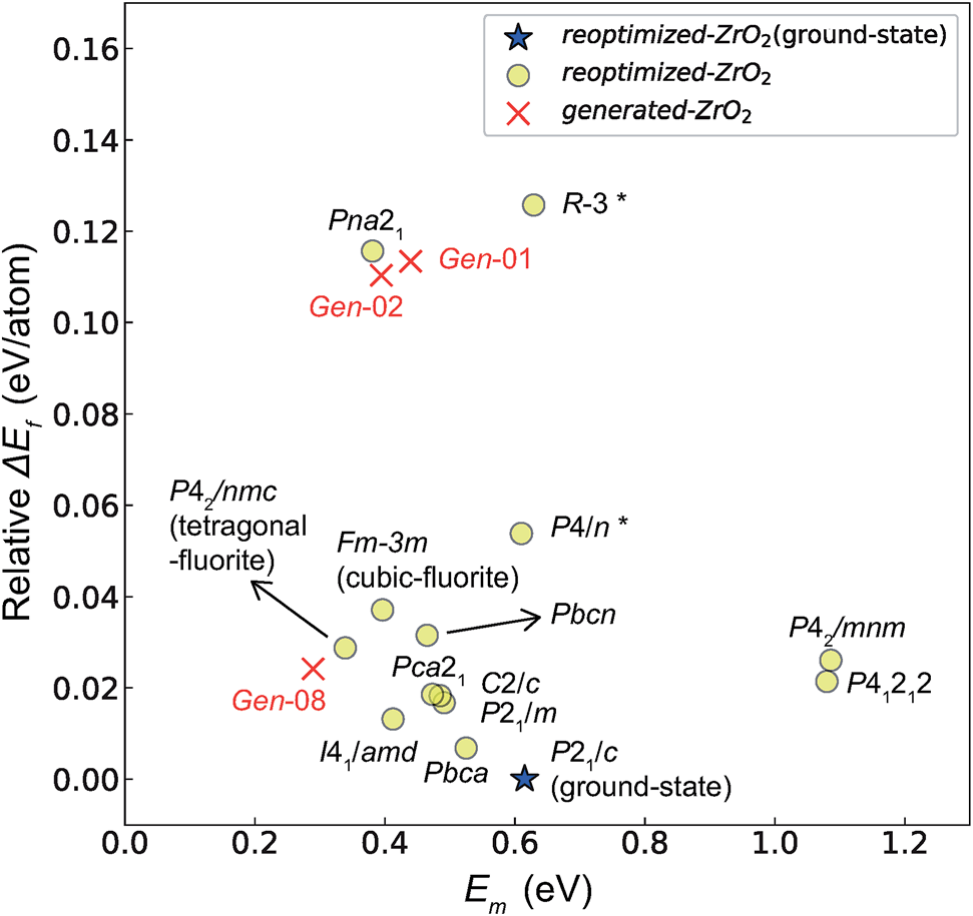
Relationship between the *E*_m_ and relative Δ*E*_f_ (compared with that of the ground-state *P*2_1_/*c* structure) of the 14 crystal structures of reoptimized-ZrO_2_ and three crystal structures of generated-ZrO_2_ which have *E*_m_ values.

Fig. 7 and 8 show the relationships between the computed *E*_v_ and *E*_m_ values, and between the relative Δ*E*_f_ (compared with that of the ground-state *P*2_1_/*c* structure) and *E*_m_ values of various ZrO_2_ crystal structures, respectively. Firstly, we compare the *E*_m_ of the 14 crystal structures of the reoptimized-ZrO_2_. The *E*_m_ values (0.39 and 0.34 eV, respectively) of the *Fm*3̄*m* and *P*4_2_/*nmc* structures are lower than that (0.61 eV) of the ground-state *P*2_1_/*c* structure. Ten crystal structures, including the *Fm*3̄*m* and the *P*4_2_/*nmc* structures, show lower *E*_m_ than that of the ground-state *P*2_1_/*c* structure.

Among these ten, eight crystal structures have only 0.05 eV per atom higher Δ*E*_f_ than the ground-state *P*2_1_/*c* structure (Table 1 and Fig. 8). This suggests that high *σ*_O_ can be realized for these structures with stabilizing methods such as extrinsic doping and the formation of epitaxial heterostructures. The *P*4_2_/*nmc* and *Fm*3̄*m* structures are confirmed to have both relatively low *E*_m_ and Δ*E*_f_. The *Pbcn* structure, which also shows relatively low Δ*E*_f_, is reportedly produced by biaxial tensile strain^53^ on fluorite-structured CeO_2_, which forms the (100) interface. This structure, obtained by 7% tensile strain, is also reported to show a higher O diffusivity than that of YSZ based on first-principles molecular dynamics calculations.^54^ The *I*4_1_/*amd*, *Pbca*, *Pca*2_1_, *C*2/*c*, and *P*2_1_/*m* structures also have the potential for stabilization, although they have somewhat larger *E*_m_ than the *P*4_2_/*nmc* and *Fm*3̄*m* structures do. These crystal structures have not been investigated as oxygen-ion conductors to the best of the authors' knowledge; however, some of them have been reported as metastable structures. The *Pbca* structure was experimentally synthesized at 600 °C and 6 GPa.^55^ This structure was also confirmed by first-principles calculations to be stabilized by hydrostatic pressure over 4.1 GPa.^56^ Mg-doped tetragonal ZrO_2_ cooled to ∼30 K reportedly transforms to the orthorhombic structure with the space group of *Pbc*2_1_ (*Pca*2_1_).^57^

The *E*_m_ values for the 14 crystal structures of the reoptimized-ZrO_2_ have similarly large *r*_p_ of 0.64 and 0.62 with the computed 
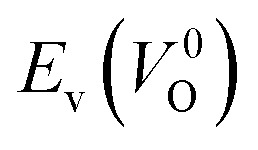
 and 
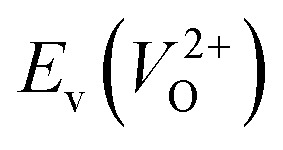
, respectively. The *E*_v_ is the energy required for breaking all the chemical bonds to separate an O atom from an oxide, and the *E*_m_ is the energy required for breaking some of the chemical bonds locally in an oxide, as shown in Fig. 2. Therefore, this correlation may be reasonable in terms of chemical bond cleavage. Correlations between *E*_v_ and *E*_m_ have also been reported for other oxides, like perovskite oxides^58^ with various kinds of constituent elements and a multinary oxide of Ba_1−*x*_Sr_*x*_Co_1−*y*_Fe_*y*_O_3−*δ*_.^59^ On the other hand, the relative Δ*E*_f_ and *E*_m_ do not show any correlation, having an |*r*_p_| of only 0.10, which implies that the relative Δ*E*_f_ is not a good single descriptor for the *E*_m_. To realize crystal structures with low values for both relative Δ*E*_f_ and *E*_m_, special challenges in processing may be necessary.

The above information suggests that we are likely to identify a low-*E*_m_ crystal structure when we initially investigate crystal structures with low *E*_v_. Therefore, for *E*_m_ calculations, we choose the four crystal structures (*Gen*-01, *Gen*-02, *Gen*-07, and *Gen*-08) of the generated-ZrO_2_ that possess the lowest *E*_v_ values in ascending order. The crystal structures between *Gen*-03 and *Gen*-06 are not chosen because we expect these structures to have properties similar to *Gen*-02 (see Fig. S4 in ESI†). The crystal structures from *Gen*-09 to *Gen*-14 are also not considered for the *E*_m_ because they may have relatively high *E*_m_ values.

The *E*_m_ values of these crystal structures from the generated-ZrO_2_ are also shown in Fig. 7 and 8. The *E*_m_ values of *Gen*-01, *Gen*-02, and *Gen*-08 are 0.42, 0.39, and 0.28 eV, respectively. Therefore, we succeed in constructing crystal structures with lower *E*_m_ as well as lower *E*_v_ than those of the ground-state *P*2_1_/*c* structure.

**Fig. 9:**
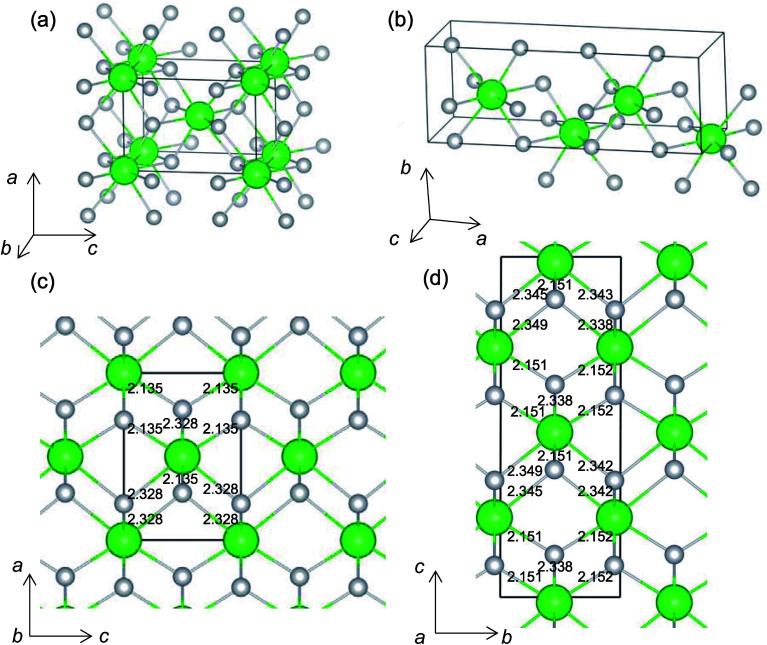
Unit-cells of (a) *P*4_2_/*nmc* and (b) *Gen*-08 structures, and supercells of (c) *P*4_2_/*nmc* and (d) *Gen*-08 structures of ZrO_2_. Numerical values in (c) and (d) are O–Zr bond lengths (in Å). Green and light gray spheres denote Zr and O atoms, respectively.

It is worth analyzing the *Gen*-08 structure further because it is very similar in crystal structure and energetic properties to *P*4_2_/*nmc*, despite its space group being *P*1. Fig. 9 shows the crystal structures of the *P*4_2_/*nmc* and *Gen*-08 structures. We confirm that *Gen*-08 and *P*4_2_/*nmc* do not become the same by structural optimizations with denser *k*-space sampling. However, the space group defined by using looser tolerance and structural properties, such as the radial distribution function and the bond lengths (see Fig. S4 in ESI†), imply that these crystal structures are quite similar; the *Gen*-08 structure is slightly distorted in terms of its bond lengths and lattice parameters (see Table S5 in ESI†). In the macroscopic view, these crystal structures may be difficult to distinguish from each other. For the construction of the *Gen*-08 structure, we only prepared the prediction model of *E*_v_ by the regression analysis and evolutionary algorithm that requires the initial structure-related input of only the number of constituting atoms of Zr and O in empty space. Therefore, the construction of *Gen*-08, which has a similar crystal structure to *P*4_2_/*nmc*, implies a successful result of a combination of evolutionary algorithm and regression analysis.

The *Gen*-07 structure, possessing an energy of 0.2 eV per atom higher than that of the ground-state *P*2_1_/*c* structure, is too unstable to contain a 
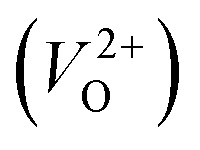
. This suggests that the energetic stability and the low *E*_v_ of the crystal structures should be considered in choosing candidates for low *E*_m_. The *Gen*-01–*Gen*-07 crystal structures have energies more than 0.1 eV per atom higher than that of ground-state *P*2_1_/*c* structure, despite having *E*_v_ lower than 5.7 eV. On the other hand, the crystal structures *Gen*-08–*Gen*-13 have less than 0.03 eV per atom higher energy than the ground-state *P*2_1_/*c* structure, although they have higher *E*_v_ values. The latter group may have higher stabilization potential than the former.

Before concluding, we note our exclusion of dynamical stabilities based on the phonon calculations with harmonic approximations^36,37^ from this study. The *P*4_2_/*nmc* and *Fm*3̄*m* structures are known to be stabilized at temperatures exceeding ∼1173 °C and ∼2370 °C, respectively. However, as Fig. S5 in ESI† shows, the *P*4_2_/*nmc* structure includes no imaginary phonon frequencies, whereas the *Fm*3̄*m* structure includes imaginary phonon frequencies despite its capacity for high-temperature stabilization, which is consistent with the phonon calculations by Wang *et al.*^60^ Therefore, anharmonic phonon effects,^61,62^ which are particularly significant at high temperatures, must be considered for these materials.

## 4. Conclusion

We have constructed a prediction model for the *E*_v_ values of various crystal structures of ZrO_2_ using a linearized RR with 11 unit-cell descriptors. The predicted *E*_v_ was used as the fitness value for crystal structure constructions based on the evolutionary algorithm in order to mainly construct crystal structures having *E*_v_ lower than that of the ground-state *P*2_1_/*c* structure. Our prediction model guarantees prediction errors of less than ∼0.15 eV for various crystal structures of ZrO_2_.

We also obtained the *E*_m_ values of various crystal structures of ZrO_2_, and found that this property is correlated with *E*_v_. On the basis of this correlation, we calculated the *E*_m_ values of several newly constructed crystal structures having low *E*_v_, and confirmed that these *E*_m_ values are also lower than those of the ground-state *P*2_1_/*c* structure and lower than or similar to those of the *P*4_2_/*nmc* and *Fm*3̄*m* structures.

We have successfully discovered various crystal structures with low *E*_v_ and *E*_m_ values for ZrO_2_ by the direct construction of crystal structures based on a combination of evolutionary algorithm and regression analysis and by reconsidering the crystal structures present in other oxides. Our DFT-unit-cell descriptors are constructed on the basis of the properties related to the chemical bonds between cations and O. Therefore, the good accuracy of the prediction model for *E*_v_ and the correlation between the *E*_v_ and *E*_m_ may be reasonable because these properties are all related to the chemical bond cleavage. We expect that the method employed in this study can be applied to determine unknown low-*E*_v_ and -*E*_m_ crystal structures of other material systems, which have not been investigated closely. In addition, the strategy described herein can be also applied to search for crystal structures with other functional properties.

## Conflicts of interest

There are no conflicts to declare.

## Supplementary Material

RA-008-C8RA02958J-s001
