# Supplementary material for: Discovery of zirconium dioxides for the design of better oxygen-ion conductors using efficient algorithms beyond data mining
Source: RSC Adv. 2018 Jul 16;8(45):25534–45. doi: 10.1039/c8ra02958j (PMC9082769; doi:10.1039/c8ra02958j)
Supplement: RA-008-C8RA02958J-s001 [file RA-008-C8RA02958J-s001.pdf]

Electronic Supplementary Material (ESI) for RSC Advances.
This journal is © The Royal Society of Chemistry 2018

Supplementary Information for *RSC Advances*

Discovery of zirconium dioxides for the design of better oxygen-ion conductors using efficient algorithms beyond data mining

Joohwi Lee[*a], Nobuko Ohba[a], and Ryoji Asahi[a]

[a]Toyota Central R&D Laboratories, Inc., Nagakute, Aichi 480-1192, Japan

* E-mail: j-lee@mosk.tytlabs.co.jp (J. Lee)

Table S1 List of excluded crystal structures which were reoptimized for ZrO_2.

Space group type	Oxide reported in Materials Project Database	Index
*Ama*2	SiO_2	Too large displacement after atomic relaxation of supercell with a V_O
$C222_1$	SiO_2	Too large displacement after atomic relaxation of supercell with a V_O
Cmce	SiO_2	Difficulty in convergence for structural optimization
Fd-3*m*	TiO_2	Too large displacement after atomic relaxation of supercell with a V_O
I-42*d*	SiO_2	Difficulty of convergence for structural optimization
*Ima*2	SiO_2	Too large displacement after atomic relaxation of supercell with a V_O
$P2_1$	SiO_2	Too large displacement after atomic relaxation of supercell with a V_O
$P3_121$	TiO_2	Too large displacement after atomic relaxation of supercell with a V_O
$P6_222$	SiO_2	Difficulty of convergence for structural optimization
$P6_322$	SiO_2	Too large displacement after atomic relaxation of supercell with a V_O
$P6_422$	SiO_2	Too large displacement after atomic relaxation of supercell with a V_O
$P6_522$	SiO_2	Too large displacement after atomic relaxation of supercell with a V_O
Pnnm	SiO_2	Difficulty in convergence for structural optimization

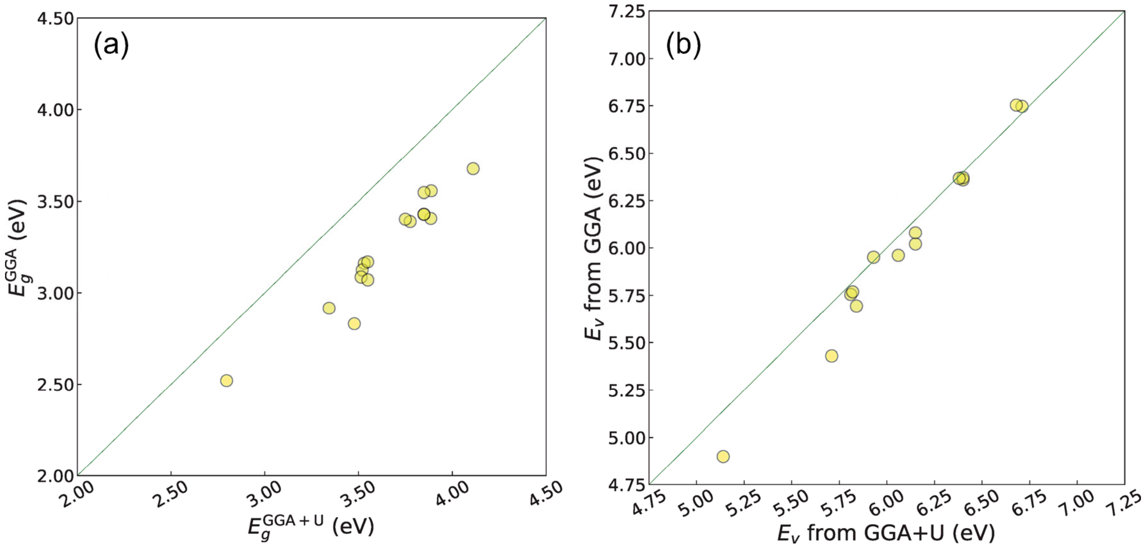

Fig. S1 Comparison of (a) E_g and (b) E_v obtained by the GGA and GGA+U methods of the 16 crystal structures of *reoptimized-*ZrO_2. The E_g^{GGA+U} are all larger than the E_g^{GGA}, whereas the E_v from the GGA+U are slightly larger or similar as the E_v from the GGA. Diagonal line denotes that the values of the GGA and GGA+U become the same.

Table S2 Chemical potential of O for the O-rich condition.

	Energy (eV/atom)	Index
From energy of O atom	−1.57	
From energy of O_2 gas molecule	−4.93	Used in this study.
From energy of O_2 gas molecule which is corrected by comparison of formation energy of various oxides between the theory and experiment	−4.25	From [Wang *et al.*, Phys. Rev. B 2006, **73**, 195107].

Table S3 Computed E_v for the 16 crystal structures of *reoptimized-ZrO_2*. Abbreviation of VBM indicates the valence band maximum. All the values are in eV.

Space group	E_g^{GGA+U}	Averaged E_v (V_O^0)	E_v (V_O^0)	E_v (V_O^{2+}) at the center of E_g	Thermodynamic transition level ε(2+/0) [a]
$P2_1/c$	3.53	6.15	6.16	4.52	2.59
			6.13	5.32	2.17
Pbca	3.52	6.15	6.14	4.50	2.58
			6.15	5.43	2.13
$I4_1/amd$	3.89	6.40	6.40	5.03	2.63
$P2_1/m$	3.85	6.40	6.41	5.35	2.46
			6.38	4.98	2.62
$C2/c$	3.85	6.38	6.38	4.89	2.67
$Pca2_1$	3.78	5.93	5.93	5.02	2.35
			5.93	4.80	2.46
$P4_12_12$	3.51	6.71	6.71	5.54	2.35
$P4_2/mnm$	3.55	6.68	6.68	5.56	2.34
Pnma	4.11	6.06		NA [b]	NA [b]
$P4_2/nmc$	3.89	5.81	5.81	3.41	3.14
Pbcn	3.85	5.82	5.82	3.62	3.03
Fm-3*m*	3.38	5.84	5.84	3.63	2.85
$P4/n$	3.34	5.71	5.80	3.15	3.00
			5.35	3.42	2.64
			5.71	4.12	2.47
			5.67	3.96	2.53
$Pna2_1$	2.80	5.14	5.30	3.48	2.31
			4.97	3.64	2.07
R-3	3.75	5.93	5.87	4.07	2.78
			6.00	4.20	2.78
$P6_3mc$	3.75	6.51		NA [b]	NA [b]

[a] If the image charge correction for the charged V_O are performed, the location of transition levels may become deeper because the E_v for the V_O^{2+} at the VBM are mostly underestimated without the correction [Kumagai *et al.*, Phys. Rev. B 2014, **89**, 195205]. However, the correction may not affect the fact that the transition levels of ε(2+/0) are deep; it is known that the $E_v(V_O^{2+})$ in the *Fm*-3*m* structure of ZrO_2 can be corrected with ~0.25 eV increased value when the dielectric constant of ~37 is used [Liu *et al.*, Comput. Mater. Sci. 2014, **92**, 22].

[b] Not applicable.

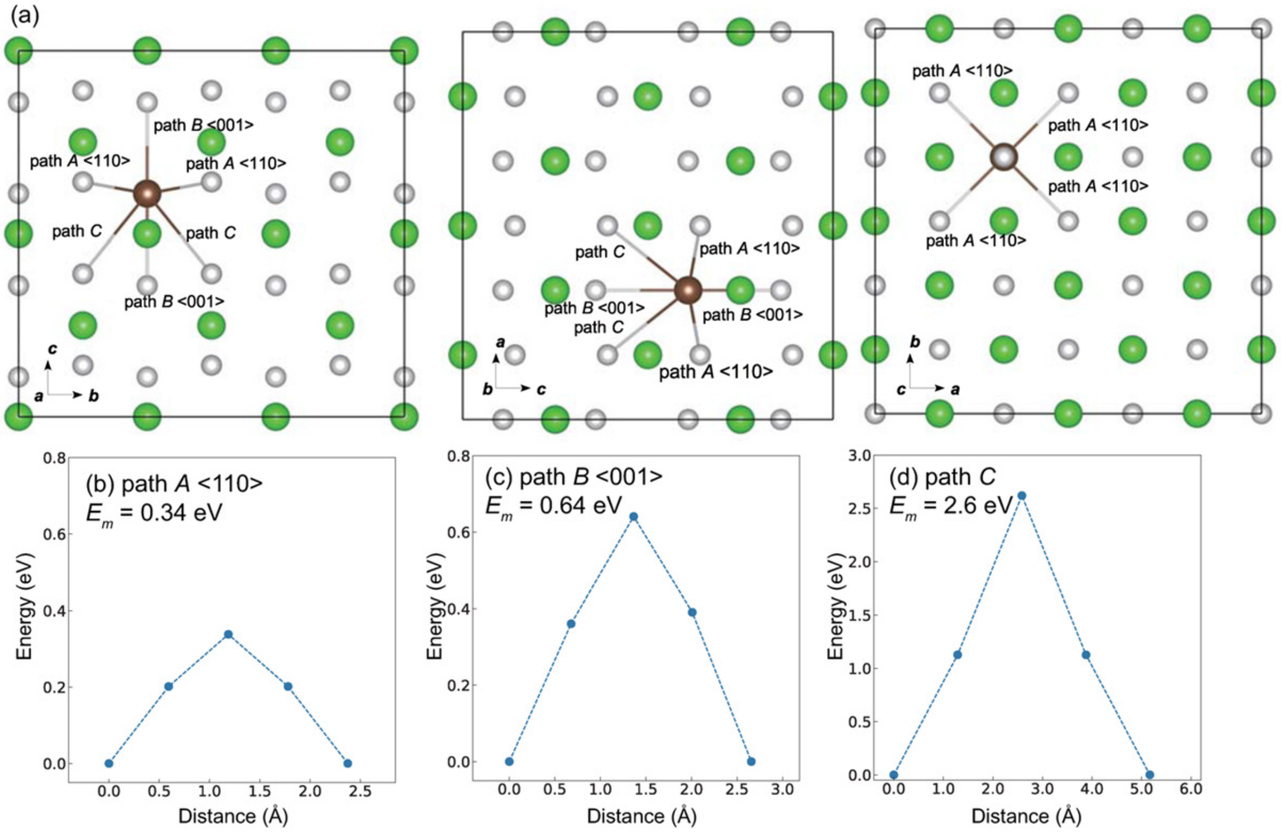

Fig. S2 (a) Three types of migration paths for the V_O to migrate to the nearest-neighboring O sites are considered with the cutoff radius of 3.5 Å. As an example, $P4_2/nmc$ structure of ZrO_2 is shown. Relative energies of the intermediates images (states) for a migration of V_O on the (b) path *A* <110>, (c) path *B* <001>, and (d) path *C*. Among three types of the migration paths, the lowest E_m of this structure is 0.34 eV on the path *A*.

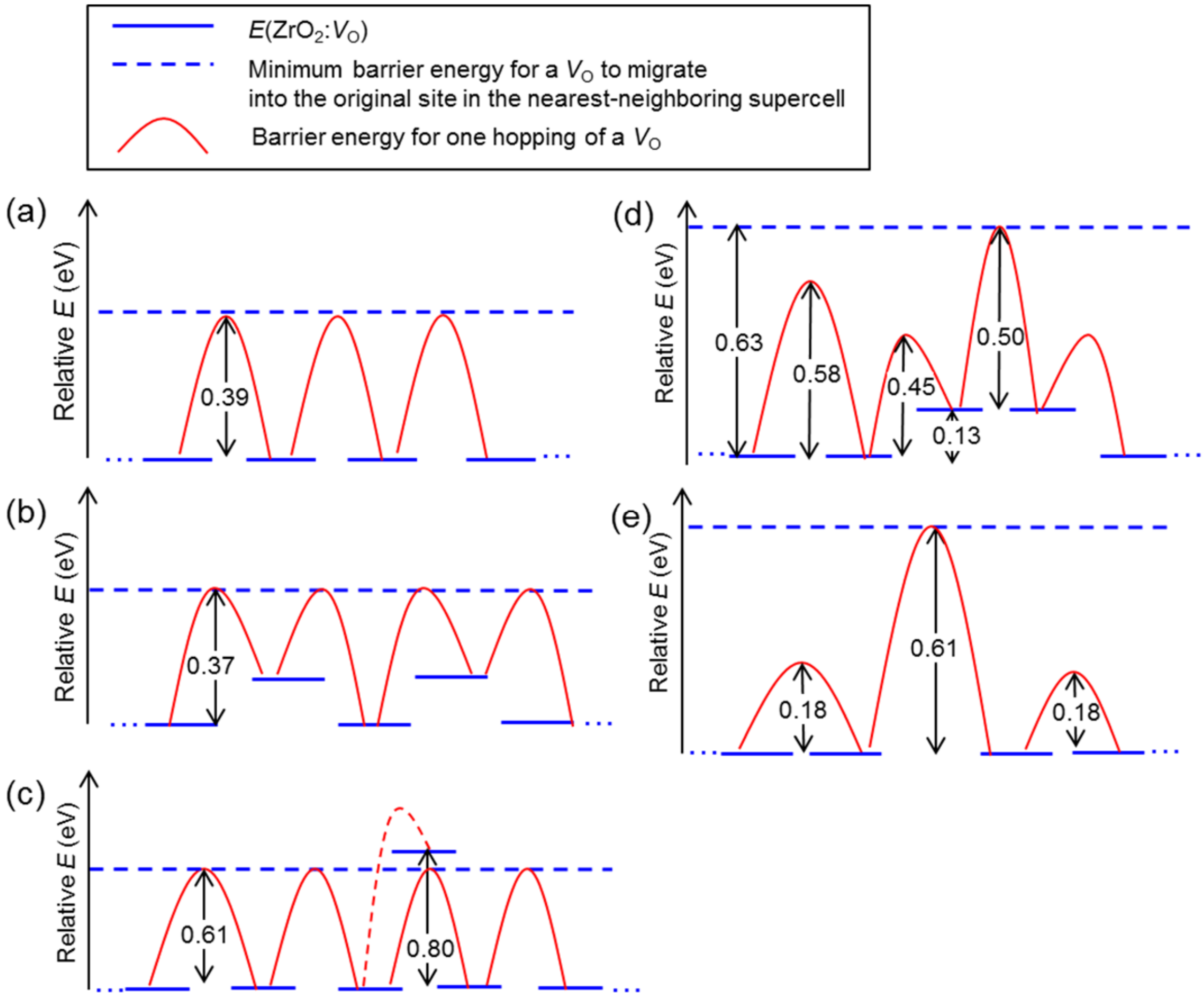

Fig. S3 Pattern of energy diagram for the minimum barrier energy for a V_O to migrate into the original site in the nearest-neighboring supercell from the examples of the (a) *Fm*-3*m*, (b) *Pna*2$_1$, (c) *P*2$_1$/*c*, (d) *R*-3, and (e) *P*4/*n* structures of ZrO_2. In cases of (a)−(c), a V_O can migrate into the original site in the nearest neighboring supercell with only one type of migration. In cases of (d) and (e), a V_O should migrate into the original site in the nearest-neighboring supercell with different types of migrations. The numerical values are in eV.

Table S4 Additional description for the pattern of the minimum barrier energy which is shown in Fig. S3.

Pattern	# of types of O sites	Crystal structures	Description [a]
Pattern (a)	1	$I4_1/amd$, $C2/c$, $P4_12_12$, $P4_2/mnm$, $P4_2/nmc$, $Pbcn$, Fm-3m	The V_O at an O-*I* site can hop between the O-*I* sites with one kind of barrier energy. (O-*I*→O-*I*→O-*I*→O-*I*→O-*I*...)
Pattern (b)	≥ 2	$Pna2_1$	The V_O at an O-*I* site can hop between O-*I* and O-*II* sites alternatively with one kind of barrier energy. (O-*I*→O-*II*→O-*I*→O-*II*→O-*I*...)
Pattern (c)	≥ 2	$P2_1/c$, $Pbca$, $P2_1/m$, $Pca2_1$	The V_O at an O-*I* site can hop between the O-*I* sites with one kind of barrier energy because this barrier energy is lower than the lowest barrier energy between O-*I* and O-*II* sites (O-*I*→O-*I*→O-*I*→O-*I*→O-*I*...)
Pattern (d)	≥ 2	*R*-3, *Gen*-01, *Gen*-02, *Gen*-08	The V_O should hop between two different kinds of V_O sites with at least two different kinds of barrier energy. (O-*I*→O-*I*→O-*II*→O-*II*→O-*I*...)
Pattern (e)	≥ 2	$P4/n$	The V_O at an O-*I* site should hop between the O-*I* sites with at least two different kinds of barrier energy. (O-*I*→(by $E^m{}_1$)→O-*I*→(by $E^m{}_2$)→O-*I*→(by $E^m{}_1$)→O-*I*..., $E^m{}_1 < E^m{}_2$)

[a] O-*I* is a V_O site with the lowest energy, and O-*II* is another site with the second lowest energy.

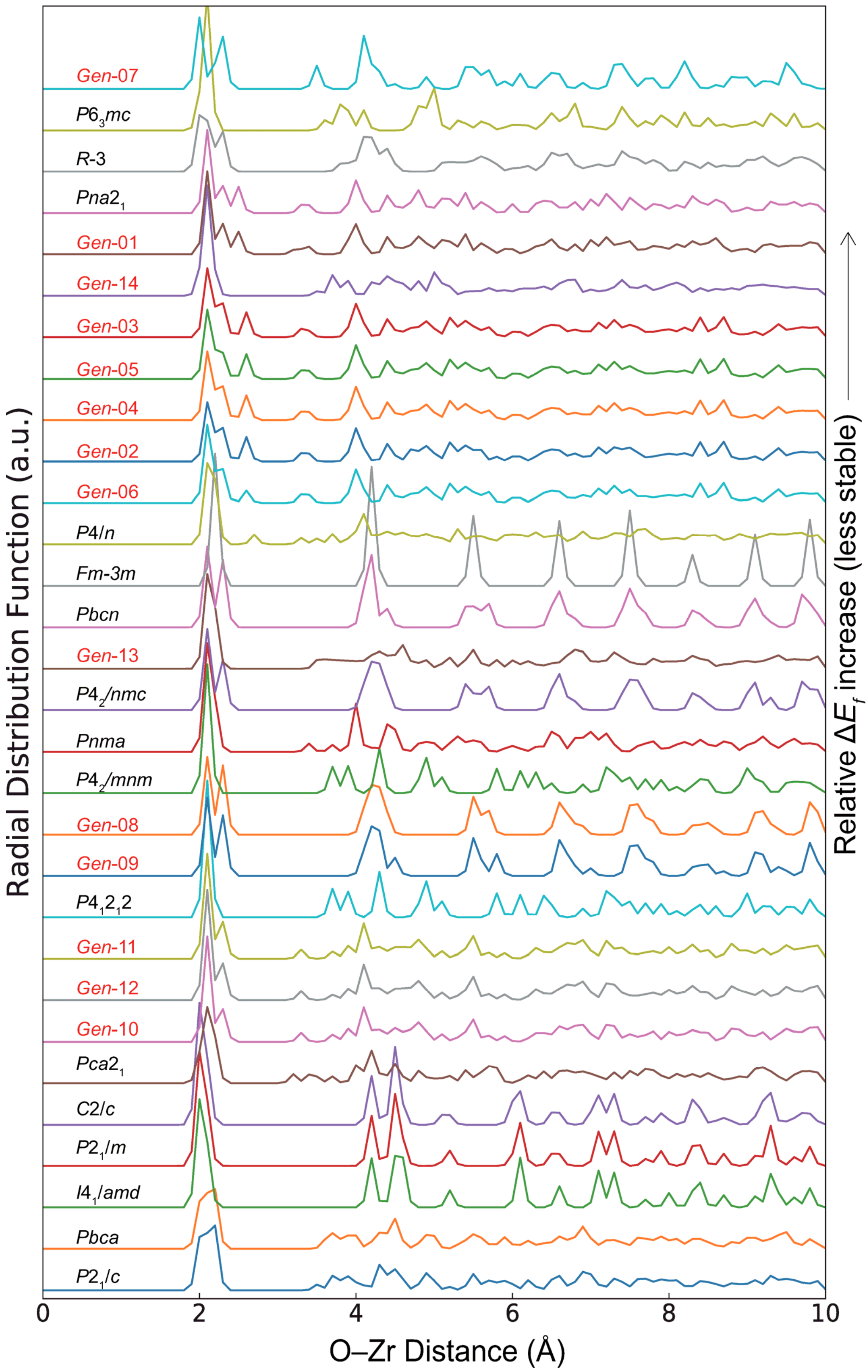

Fig. S4 Radial distribution functions according to O−Zr distances of the 30 crystal structures from *reoptimized-*ZrO_2 and *generated-*ZrO_2. From the bottom to the top, the relative ΔE_f(compared with that of the ground-state $P2_1/c$ structure) of crystal structures increase.

Table S5 Lattice parameters of unit-cells [Fig. 9(a) and (b)] of two crystal structures of ZrO_2.

Crystal Structure	$\boldsymbol{x}$ (Å)	$\boldsymbol{y}$ (Å)	$\boldsymbol{z}$ (Å)	$\boldsymbol{\alpha}$ (˚)	$\boldsymbol{\beta}$ (˚)	$\boldsymbol{\gamma}$ (˚)	Number of atoms
$P4_2/nmc$	3.626	3.626	5.174	90.00	90.00	90.00	2 Zr, 4 O
Gen-08	10.420	3.651	3.655	90.02	89.85	90.21	4 Zr, 8 O

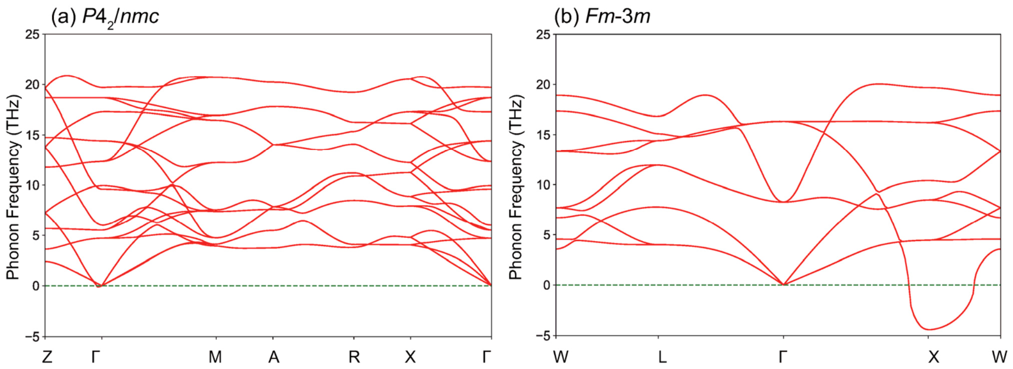

Fig. S5 Phonon dispersion curves of (a) tetragonal fluorite ($P4_2/nmc$) and (b) cubic fluorite (Fm-3m) structures of ZrO_2.
